# Case of hereditary papillary renal cell carcinoma

**DOI:** 10.3402/jchimp.v1i4.9468

**Published:** 2012-01-26

**Authors:** Sadaf Mustafa, Nima Jadidi, Sheila F. Faraj, Ronald Rodriguez

**Affiliations:** 1 Department of Internal Medicine, Union Memorial Hospital, Baltimore, MD, USA; 2 Department of Pathology, Johns Hopkins University School of Medicine, Baltimore, MD, USA; 3 Department of Urology, Johns Hopkins University School of Medicine, Baltimore, MD, USA

**Keywords:** familial papillary renal cell carcinoma, hereditary leiomyomatosis and renal cell carcinoma, met proto-oncogene, hepatocyte growth factor/scatter factor, renal cancer

## Abstract

Renal cell carcinoma is the most common type of renal malignancy and it originates from the renal tubular epithelium. Due to the diversity in the histopathological and molecular characteristics, it is typically subclassified into five different categories. Papillary renal cell carcinoma is one subclassification and it includes two variants: sporadic and hereditary. Although the hereditary form comprises a smaller number of cases of papillary renal cell carcinoma, an understanding of the molecular pathways and genetic changes continues to play a significant role in the development of new targeted therapies. Along with recommending appropriate lifestyle modification, further investigation into the molecular pathogenesis of hereditary papillary renal cell carcinoma will continue to be invaluable for the clinical management of renal cell carcinoma. In this article, we discuss a case of the hereditary papillary renal cell carcinoma along with an overview of the disease.

According to the 2011 cancer statistics compiled by the American Cancer Society annually, the incidence rate of renal cancer has increased between 2 and 3% since 1992. This trend has been attributed to earlier detection of renal cell cancer in patients as opposed to a true increase in cancer occurrence. Mortality rates have been decreasing for both genders since 2002. Evidence suggests that cigarette smoking and obesity are significant modifiable risk factors (1). Preventative efforts to curtail these risk factors will continue to play a large role in lowering the occurrence of renal cancer. The decrease in mortality of renal cancer in the past decade has been partly attributed to its earlier detection and treatment. More than half of the newly diagnosed cases are discovered at an early stage. Surgical excision of the tumor is typically the primary recommended treatment modality. The 5-year survival rate for these patients is greater than 90%. Medical management is typically reserved for locally invasive or metastatic disease. Renal cancers tend to be resistant to many standard chemotherapeutic agents as well as radiation therapy. Interferon-alpha and interluekon-2 had traditionally been the mainstay in treatment of non-resectable tumors and late-stage metastatic disease; however, their use in preventing recurrence after surgical excision has not proven efficacious. Because of our growing understanding of the molecular pathways leading to renal cancer, many new targeted therapies have been developed in the last decade.

Hereditary forms of papillary renal cancer comprise a very small portion of newly diagnosed renal cancers (2). Nevertheless, an understanding of familial renal cancer syndromes has led to greater insights into kidney tumor formation and potential therapeutic targets. This article includes a case history of a patient with hereditary papillary renal carcinoma (HPRC) and a relevant case discussion. The importance of the case lies in the early identification of this rare familial form and its timely treatment.

## Case report

**Fig. 1:**
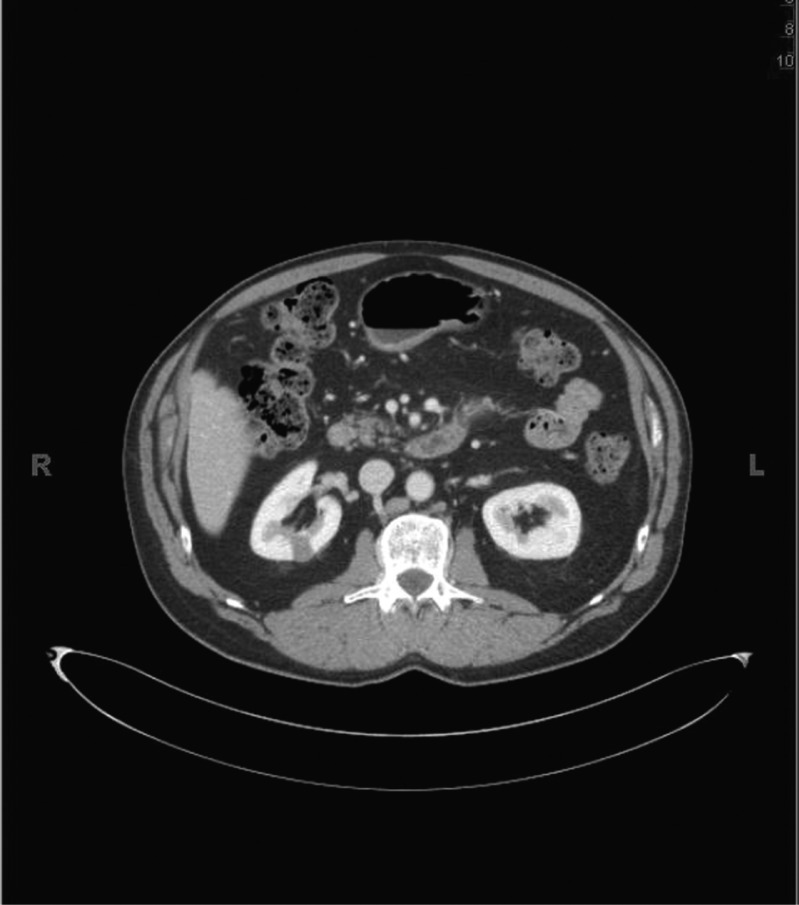
Abdominal Computed Tomography scan image of the patient with HPRC: The abdomen CT scan with contrast of the patient showing a well defined homogeneous hypodense mass of 1.3×1.9 cm in the right kidney.

**Fig. 2:**
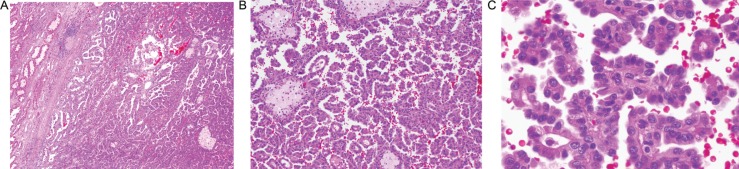
H&E histological slide of the patient's papillary renal cell carcinoma, Fuhrman grade III: Low powered magnification (50X) showing characteristic papillary architecture. Non neoplasticrenal parenchyma is seen on left (A). Occasional papillary structures demonstrate typical foamy histiocytes in fibrovascular cores upper left portion (100X) (B). High power magnification showing neoplastic papillary structures lined by eosinophilic cuboidal epithelial cells with ovoid nuclei and conspicuous nucleoli (400X) (C).

We present a case of 61-year-old Caucasian male who presented with a history of right renal mass that was discovered incidentally 5 years ago. Since then he was followed by serial CT scans annually. On these scans, it appeared to be a minimally enhancing exophytic mass of less than 2 cm in largest diameter in the posterior upper pole of the right kidney. The patient had no symptoms and was otherwise in a good state of health. His family history was remarkable for renal neoplasm; his mother died of metastatic RCC with an unknown histology, whereas his brother and sister had Fuhrman grade III PRC carcinoma. There was no personal or family history of cutaneous leiomyomatosis or leiomyosarcoma. His repeated CT scan showed a well-defined homogeneous hypodense 1.3×1.9 cm minimally enhancing lesion in the posterior upper pole of the right kidney and 1 mm indeterminate lesion in the left kidney with no evidence of local or distant metastasis (Fig. 1). In light of the strong family history, HPRC was considered. The patient underwent open partial nephrectomy of the right kidney with no intraoperative complications. The right renal mass and frozen sections from the margins of the resection were sent for histopathology that showed a unifocal mass of 2 cm in diameter bearing PRC histology with Fuhrman grade III (Fig. 2) The margins were negative, and there was no evidence of invasion of the surrounding structures. The patient had an unremarkable postoperative course and is being followed every 6 months with complete blood count, complete metabolic panel, and serial imaging studies. Surveillance will continue throughout his life due to the recurrence risk.

## Discussion

In 1989, Kovacs proposed that PRC should be considered as a separate entity of renal neoplasms and suggested that a renal tumor should be classified as such if at least 75% of the tumor consists of papillary structures (3). This histopathological classification was modified by Delahunt and Eble who further subcategorized PRC as type1 and type2 that account for 5 and 10% of renal cancers, respectively (4). Generally, type 2 PRC has a larger tumor size, higher tumor stage, and nuclear grade than type1 and thus follows a relatively more aggressive course. The key histological features that differentiate the two types are as follows:

1. PRC type1 is characterized by the presence of short papillae covered by small cuboidal cells that have pale cytoplasm and small oval nuclei with barely visible nucleoli. Additionally frequent glomeruloid papillae, papillary edema, foamy macrophages in papillary cores, and psammoma bodies may also be present (5).
2. PRC type2 shows histologic features that consist of papillae covered by large cells with abundant eosinophilic cytoplasm and large spherical nuclei with prominent nucleoli.

PRC can occur in both sporadic and hereditary forms. As outlined in Table 1, there are two well recognized familial forms of PRC; hereditary papillary renal carcinoma (HPRC) and hereditary leiomyomatosis and renal cell carcinoma. In 1994, Zbar et al. described HPRC as an autosomal dominant inherited form of PRC, which is characterized by a tendency to develop multiple, bilateral papillary renal tumors (6). It was in the late 1990s that Schmidt and colleagues mapped the HPRC gene on chromosome 7q31–34 and identified activating mutations in the tyrosine kinase (TK) domain of *met* proto-oncogene (7). The *met* gene encodes for a transmembrane TK receptor also known as hepatocyte growth factor/scatter factor (HGF/SF) receptor or c-Met, which is overexpressed in the tumors with PRC type1 histology. The mutations are present on the extracellular domain of the receptor where the only known natural ligand, the HGF/SF, interacts with the receptor. The activation of intrinsic TK is essential for the HGF/MET pathway to promote cell growth, motility, and proliferation; it has an important role in tissue repair and regeneration. The mutations found in *met* gene interfere with the autoinhibition of TK and in fact facilitate its conversion to the more active form by lowering the threshold for receptor activation, stabilizing the active conformation of the kinase, and in some cases making it less susceptible for inactivation by phosphatases (8). Interestingly, only 13% of the patients with sporadic PRC have this mutation and they show the same histologic features as that of HPRC (9). Due to the slow progression of disease, the age at presentation usually lies between the fourth and sixth decades of life. Clinical symptoms may vary from incidental diagnosis to a more advanced disease with hematuria, abdominal pain, and abdominal mass. The patients with HPRC have bilateral, multiple, and multifocal renal tumors bearing type1 PRC histology, and microscopically up to 3,400 tumors may be identified in a single kidney (10). No extrarenal manifestations have been identified in these patients. CT scan with intravenous contrast is preferred over ultrasound as the main modality for diagnosis and follow-up. These tumors are often mistaken for renal cysts on CT scan as they are typically hyperdense and have a hypovascular nature. The treatment options may include close observation in those cases where the mass is less than 3 cm in largest diameter to nephron sparing surgery or partial nephrectomy with larger tumor sizes. Cryoablation and minimally invasive radiofrequency ablation may be used as an alternative for small and/or multiple tumors (2). Several MET kinase inhibitors such as ARQ197 and Foretinib have been developed and are undergoing testing. Foretinib, an oral dual-kinase agent, targets TK domain of MET and VEGFR2 and is currently being evaluated in an ongoing multicenter phase II clinical trial for the treatment of sporadic PRC with met mutations and HPRC that meet certain criteria.

**Table 1:** Hereditary forms of Papillary Renal Cell Carcinoma

Hereditary forms	Genetic abnormality	Histopathology	Clinical Manifestations
HPRC	HPRC gene Mutations in the tyrosine kinase (TK) domain of *met* proto-oncogene	Short papillae covered by small cuboidal cells with pale cytoplasm, small oval nuclei and hardly visible nucleoli.	No manifestations other than papillary renal cell carcinoma
HLRCC	Mutations in the HLRCC gene resulting in Fumarate Hydratase deficiency	Papillae covered by large cells that have abundant eosinophilic cytoplasm, large spherical nuclei and prominent nucleoli.	Cutaneous leiomyomatosis, uterine leiomyomata or leiomyosarcoma occurring in association with Papillary Renal cell carcinoma

An overview of the two hereditary forms of papillary renal cell carcinoma (PRC). Hereditary Papillary Renal Cell carcinoma (HPRC) and Hereditary leiomyomatosis and Renal cell Carcinoma (HLRCC) bearing type1 and type2 PRC histology respectively. They are characterised by the presence of distinct genetic mutations that makes HLRCC the most aggressive form. The clinical manifestations of HPRC and HLRCC vary depending upon the disease severity at the time of presentation; additionally a patient with HLRCC may also have history of Leiomyomas, which are not present in HPRC.

Despite the complexities inherent in investigating cancers at the molecular level, a better understanding may be essential for more individualized therapy. Immunotherapies such as interferon-alpha and interleukin-2 have been the main treatment options when medical management is warranted; however, improved understanding of the biology of renal cancers has led to the development of new targeted therapies that block the tumor's blood supply or disrupt other parts of renal cancer cells. Several agents have been approved by the FDA and many more are undergoing clinical trials. These agents have given clinicians many more therapeutic options for patients with late stage disease. A continued effort to understand the molecular pathways leading to the different renal cancer types will result in further novel approaches to both treating renal cancer and stopping its recurrence in postsurgical patients. Clinicians should have high index of suspicion, if there is a strong family history of renal cancers. Prompt intervention should be offered to the patient and screening to the first-degree relatives. Genetic testing should also be offered to the patient and the families after appropriate counseling. Early identification and intervention in a timely manner may provide more therapeutic options and reduce the morbidity associated with renal cell carcinoma.
